# Mixed Adenoneuroendocrine Carcinomas (MANECs) of the Gastrointestinal Tract: An Update

**DOI:** 10.3390/cancers4010011

**Published:** 2012-01-16

**Authors:** Stefano La Rosa, Alessandro Marando, Fausto Sessa, Carlo Capella

**Affiliations:** 1 Department of Pathology, Ospedale di Circolo, viale Borri 57, 21100 Varese, Italy; 2 Department of Surgical and Morphological Sciences, University of Insubria, via O. Rossi 9, 21100 Varese, Italy; E-Mails: alessandromarando@gmail.com (A.M.); fausto.sessa@uninsubria.it (F.S.); carlo.capella@ospedale.varese.it (C.C.)

**Keywords:** adenoneuroendocrine carcinoma, mixed exocrine-neuroendocrine carcinoma, gut

## Abstract

The systematic application of immunohistochemical techniques to the study of tumors has led to the recognition that neuroendocrine cells occur rather frequently in exocrine neoplasms of the gut. It is now well known that there is a wide spectrum of combinations of exocrine and neuroendocrine components, ranging from adenomas or carcinomas with interspersed neuroendocrine cells at one extreme to classical neuroendocrine tumors with a focal exocrine component at the other. In addition, both exocrine and neuroendocrine components can have different morphological features ranging, for the former, from adenomas to adenocarcinomas with different degrees of differentiation and, for the latter, from well differentiated to poorly differentiated neuroendocrine tumors. However, although this range of combinations of neuroendocrine and exocrine components is frequently observed in routine practice, mixed exocrine-neuroendocrine carcinomas, now renamed as mixed adenoneuroendocrine carcinomas (MANECs), are rare; these are, by definition, neoplasms in which each component represents at least 30% of the lesion. Gastrointestinal MANECs can be stratified in different prognostic categories according to the grade of malignancy of each component. The present paper is an overview of the main clinicopathological, morphological, immunohistochemical and molecular features of this specific rare tumor type.

## 1. Introduction

The first description of a gastrointestinal tumor with an exocrine and a neuroendocrine component was published by Cordier in 1924 [1]. Since then, several cases have been reported using many different names including composite carcinoid, mucin-producing carcinoid, argentaffin cell adenocarcinoma, goblet cell carcinoid, adenocarcinoid, small cell undifferentiated carcinoma, and so on. In 1987, Lewin suggested classifying such neoplasms into three different subtypes: collision tumors, combined tumors, and amphicrine tumors [2]. The use of all these different names led to considerable confusion among clinicians, surgeons, gastroenterologists and pathologists. In the 2000 WHO classification of endocrine tumors, such neoplasms were defined as mixed exocrine-endocrine tumors when each component represents at least 30% of the lesion [3]. In the most recent WHO classification of neoplasms of the gastrointestinal tract, such neoplasms are called “mixed adenoneuroendocrine carcinomas” (MANECs) [4].

**Figure 1:**
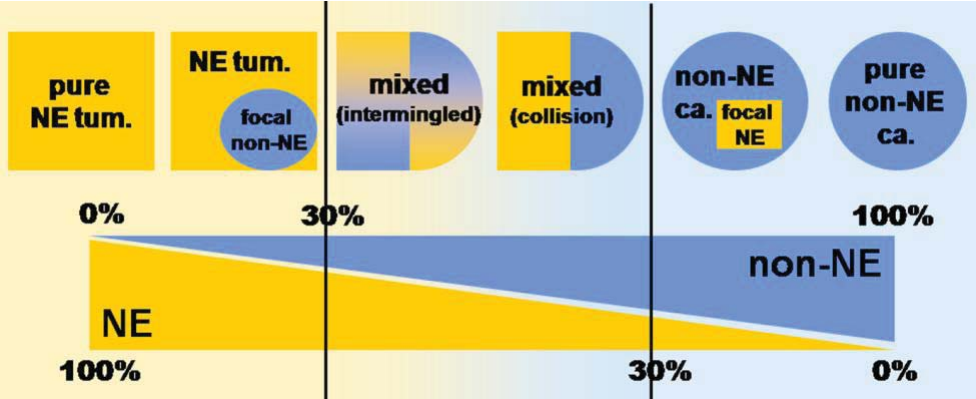
Schematic representation showing the wide spectrum of combinations of exocrine and neuroendocrine components in human tumors, ranging from neuroendocrine neoplasms with a focal exocrine component at one extreme (left) to exocrine carcinomas with interspersed neuroendocrine cells at the other (right). However, mixed exocrine-neuroendocrine tumors (middle) are only those neoplasms in which each component represents at least 30% of the lesion. NE: neuroendocrine; tum.: tumor; ca.: carcinoma (modified from Volante *et al*. [6]).

The presence of a neuroendocrine component in gastrointestinal adenomas/adenocarcinomas has often been reported. Indeed, the systematic application of immunohistochemical techniques to the study of gastrointestinal tumors has demonstrated that neuroendocrine cells occur rather frequently in non-endocrine neoplasms. Similarly, the presence of an exocrine component in gastrointestinal neuroendocrine neoplasms, especially in high grade neuroendocrine carcinomas, has also been widely documented. There is a wide spectrum of such combinations of exocrine and neuroendocrine components (Figure 1), ranging from adenomas or carcinomas with interspersed neuroendocrine cells on the one end to classical neuroendocrine tumors with a focal exocrine component on the other end [5,6]. 

**Figure 2:**
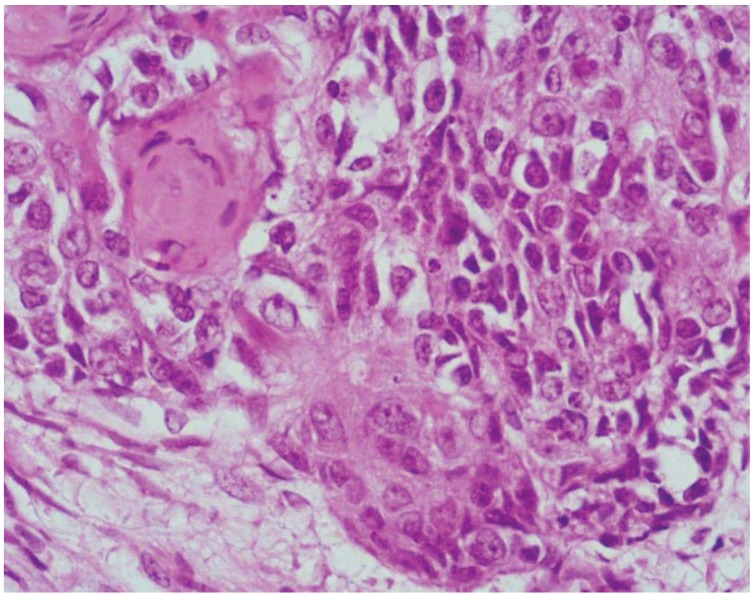
Ano-rectal NEC showing a focal squamous differentiation characterized by groups of squamous cells with eosinophilic cytoplasm.

In addition, both the exocrine and the neuroendocrine components can have different morphological features ranging from adenomas to adenocarcinomas with different degrees of differentiation in exocrine components and from well differentiated to poorly differentiated neuroendocrine tumors in neuroendocrine components [5]. However, although this spectrum of combinations is frequently observed in routine practice, mixed exocrine-neuroendocrine tumors are rarely found. By definition, such neoplasms are those in which each component represents at least 30% of the lesion [3,4]. In the 2010 WHO classification of tumors of the digestive tract, mixed exocrine-neuroendocrine carcinomas are defined as mixed adenoneuroendocrine carcinomas (MANECs) [4]. They are morphologically recognizable as both gland forming epithelial and neuroendocrine neoplasms and they are defined as carcinomas since both components are histologically malignant. An exocrine component of squamous cell carcinoma, although very rare, can also be observed, especially in esophageal and anal tumors (Figure 2).

**Figure 3:**
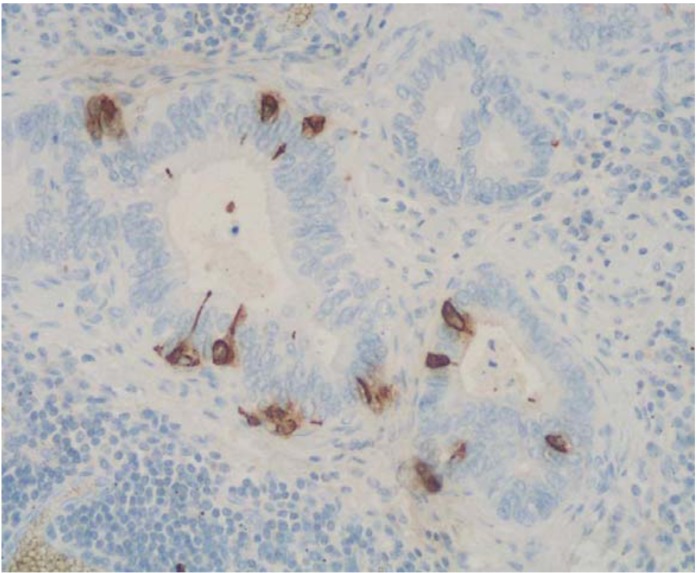
An adenocarcinoma showing scattered neuroendocrine cells, demonstrated using anti-chromogranin A antibody, cannot be classified as a MANEC.

It is worth noting that adenocarcinomas with scattered neuroendocrine cells (Figure 3), shown by immunohistochemistry, cannot be categorized as MANECs, nor can neuroendocrine neoplasms with a focal non-neuroendocrine component. Although goblet cell carcinoids of the appendix have been traditionally considered as mixed exocrine-neuroendocrine neoplasms, in the 2010 WHO classification of tumors of the digestive system they have been described in both adenocarcinoma and neuroendocrine tumor sections [7,8], giving rise to some confusion. Since in most cases the neuroendocrine component does not reach 30% and is mostly represented by scattered neuroendocrine cells, we do not report a detailed description of such peculiar neoplasm in this review. Appendiceal tubular carcinoids, although able to produce mucins focally, are classified among neuroendocrine neoplasms in the 2010 WHO classification [7] and for this reason we do not include them in the present paper. However, it is worth noting that these particular appendiceal neoplasms require further investigation to better clarify their histogenesis, molecular profile, and, consequently, classification.

In some MANECs the neuroendocrine and exocrine components occur in separate areas of the same lesion (composite or collision neoplasms), while in other MANECs they are intimately and diffusely admixed (combined neoplasms). In amphicrine tumors exocrine and neuroendocrine features are present in the same neoplastic cell, which shows a divergent immunophenotype.

In addition to these MANEC types in which both components are histologically malignant, very rare tumors composed of adenoma and well differentiated neuroendocrine tumor (NET according to the nomenclature proposed by the 2010 WHO classification) have been also described in the colon-rectum [9,10]. In the 2010 WHO classification of tumors of the digestive system, these mixed adenoma-NET neoplasms were not specifically categorized [4]. Due to their low biological aggressiveness and their peculiar morphological features, we suggest using the term of mixed adenoneuroendocrine tumor (MANET) to diagnose these peculiar mixed neoplasms. This term, although not included in the 2010 WHO classification, would be more appropriate because it underlines the better prognosis of this tumor category (see below). However, it must be underlined that, in spite of their mild to moderate nuclear atypia and low number of mitoses, MANETs can metastatize.

The clinical significance and the influence on survival of focal neuroendocrine differentiation in gut adenocarcinomas still remain controversial. Conversely, gastrointestinal MANECs can be grouped in different prognostic categories according to the grade of malignancy of each component [5]. A provisional prognostic classification of gastrointestinal MANECs is provided in Table 1.

MANECs may constitute a diagnostic challenge because frequently only one component of the neoplasm is identified. This leads to an incomplete diagnosis and suboptimal treatment.

## 2. Mixed Adenoneuroendocrine Carcinomas (MANECs)

### 2.1. High Grade Malignant MANEC

**Figure 4:**
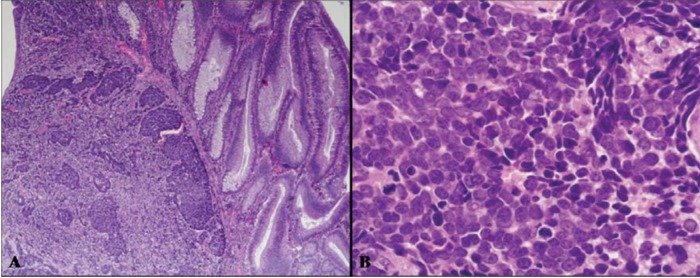
(**A**) is an example of a colonic high grade MANEC composed of an adenoma (right) and of a small-intermediate cell NEC invading the bowel wall (left). The small-intermediate cell NEC is composed of atypical small cells with scarce cytoplasm and hyperchromic nuclei lacking nucleoli. High mitotic index is observed (**B**).

**Figure 5:**
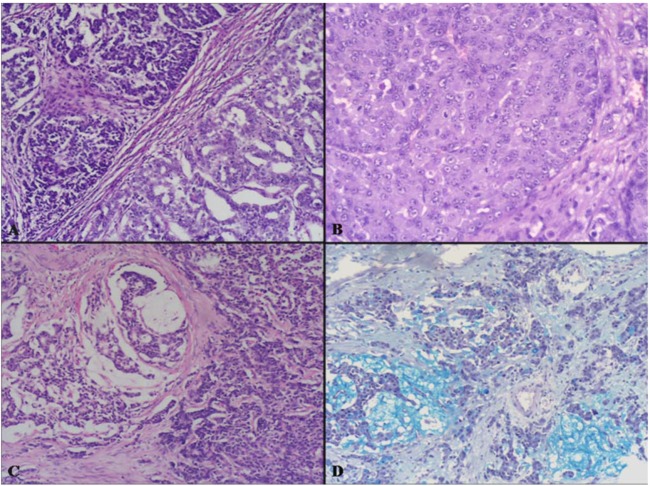
The exocrine component of high grade MANECs can be represented by tubular adenocarcinoma (**A**) or adenocarcinoma with obvious mucin secretion (**C**). The neuroendocrine component can be represented by a large cell NEC showing large cells with abundant eosinophilic cytoplasm and nuclei with dispersed chromatin showing evident nucleoli (**B**). Adenocarcinomas with obvious mucin secretion showing abundant mucinous deposits stained with the alcian blue-PAS staining (**D**).

This is a highly malignant composite or combined neoplasm formed by an adenomatous (villous or tubulo-villous) or carcinomatous (adenocarcinoma or squamous cell carcinoma) component and by a poorly differentiated (small, intermediate or large cell type) neuroendocrine carcinoma (NEC) (Figure 4 and Figure 5). This neoplasm has been reported in the esophagus [11,12,13,14,15], stomach [16,17,18,19,20,21], ampullary region [22], large bowel [23,24,25,26,27,28,29,30,31], and anorectal region [32,33].

**Table 1:** Types of mixed exocrine-neuroendocrine neoplasms of the gastrointestinal tract, grouped according to the grade of malignancy.

**Mixed Adenoneuroendocrine Carcinoma (MANEC)**
*High grade malignant*
Mixed adenoma/adenocarcinoma-NEC
*Intermediate grade malignant*
Mixed adenocarcinoma-G1/G2 NET ^*^
Amphicrine carcinoma
**Mixed Adenoneuroendocrine Tumor (MANET)**—*Provisional category*
*Low grade malignant*
Adenoma-NET

NEC: poorly differentiated neuroendocrine carcinoma; ^*^: G1-G2 according to WHO 2010 classification [4]; NET: neuroendocrine tumor.

High grade MANECs with a small cell or large cell NEC component are rare. Approximately 100 documented cases appear in the literature. Most high grade MANECs of the esophagus occur in the distal half [14,34], while those of the stomach show equal distribution in the upper and lower part [21,35,36]. High grade MANECs can also arise in either the right or the left colon [23,24,30,31].

**Figure 6:**
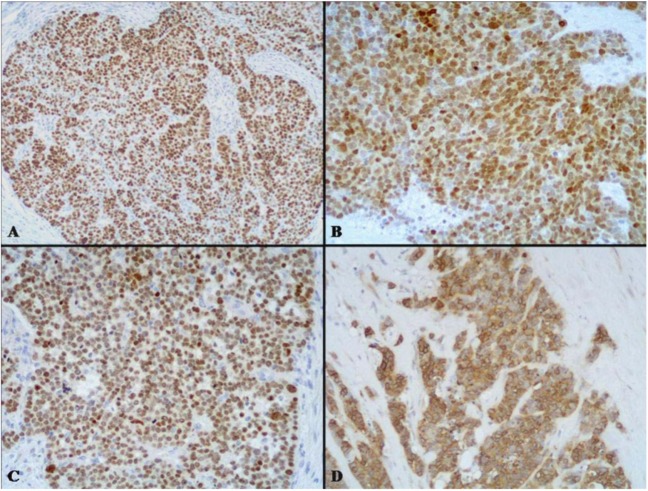
The neuroendocrine component of high grade MANECs generally shows p53 nuclear immunoreactivity (**A**). In addition, CDX2 (**B**) and TTF1 (**C**) nuclear positivity can be also demonstrated in neoplastic cells. CD117 immunoreactivity can be observed in some cases and its expression has been recently demonstrated to be associated with worse patient outcome in colorectal MANECs (**D**).

Macroscopically, these neoplasms, independently of the site of origin, appear as polypoid masses or ulcerating stenotic lesions measuring from 0.5 to 14 cm in greatest diameter, with a mean size of about 5 cm. Histologically, the NEC component is morphologically similar to small cell or large cell NEC of the lung and corresponds to a grade 3 neuroendocrine neoplasm, according to the 2010 WHO classification [4]. The small cell component has a diffuse or nesting growth pattern and is formed by small to intermediate sized cells with scanty cytoplasm and fusiform nuclei with granular chromatin and inconspicuous nucleoli (Figure 4). Diffuse geographic necrosis is common. The mitotic rate is high ranging from 20 to 80 mitotic figures per 10 high-power fields. The large cell NEC, or high grade NEC (HGNEC) non small cell type [15], component is formed of cells with an abundant cytoplasm showing more vesicular nuclei with prominent nucleoli as well as a more prominent organoid, trabecular and palisading pattern (Figure 5). Immunohistochemically, pure neuroendocrine areas of both small and large cell neuroendocrine components are diffusely positive for synaptophysin and usually for chromogranin A, although to a lesser extent [31,37,38]. At least two out of three commonly used neuroendocrine markers (synaptophysin, chromogranin A or CD56) must be abundantly expressed to formulate a diagnosis of high grade MANEC [4]. The Ki67 labeling index is usually very high (60%–90%) [12]. Expression of appropriate or inappropriate hormonal peptides, such as somatostatin, adrenocorticotropic hormone (ACTH) or vasoactive intestinal peptide (VIP), have been detected in a few cases of MANEC [39,40,41]. p53 nuclear accumulation (Figure 6A) was detected in 7/11 of our gastric, 3/3 ampullary, and 12/12 colorectal cases [21,31]. In colorectal MANECs neuroendocrine cells generally show nuclear immunoreactivity for CDX2, especially in the large cell subtype (Figure 6B). In addition, TTF1 (Figure 6C) and ASH1 immunoreactivity has also been documented in some neoplasms [31]. The expression of these transcription factors does not show a prognostic significance and they cannot be used as site-related markers because they have also been found to be expressed in NECs of other sites such as the lung, gallbladder and urogenital system [31,42,43]. However, their expression is interesting because it indicates the phenotypical heterogeneity of the neuroendocrine component of colorectal MANECs. Like pure colorectal NECs, colorectal MANECs showing CD117 immunoreactivity (Figure 6D) and vascular invasion were associated with worse patient survival [31]. Ultrastructural analysis reveals a few small (100 to 200 nm in diameter) neurosecretory granules resembling those of immature “protoendocrine” cells of early fetal development [44].

The non-neuroendocrine component of high grade MANECs can be represented by tubulo-villous or villous adenoma [21,23,24,25,30,31], by adenocarcinoma [11,14,15,27,30,31,39,45] or, more rarely, by squamous cell carcinoma [11,12,15,31]. The squamous cell component is most often observed in esophageal and anorectal MANECs [11,12,15], whereas adenomatous or adenocarcinomatous areas are prevalently detected in gastric [17,19,21,35] and colorectal MANECs [15,23,24,25,30,31].

**Figure 7:**
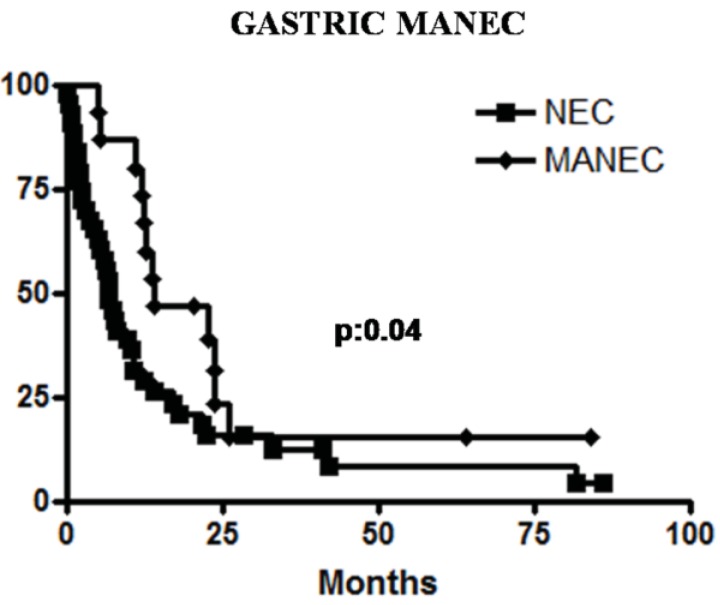
Patients with gastric MANECs show a better survival than patients with gastric NECs in our series.

Prognosis of high grade MANECs largely depends on stage and tumor type. In a recent retrospective series of esophageal NECs and MANECs, better survival was registered for patients with loco-regional disease compared to patients with distant metastases [14]. In general, patients with gastrointestinal MANECs seem to have a better median overall survival than patients with pure NECs and this seems to be mainly due to the higher stage at the time of diagnosis of the latter [14,15]. A similar prognostic behavior was registered in our cases of high grade gastric MANECs (Figure 7) [21]. Conversely, in a recent investigation of colorectal MANECs we did not observe a different survival rate between patients with MANECs compared to patients with pure NECs [31], suggesting that some clinical differences between NECs and MANECs may be site-related [46].

### 2.2. Intermediate Grade Malignant MANEC

In this category two different tumor types are included: mixed adenocarcinoma-neuroendocrine tumor and amphicrine carcinoma. In the first category the exocrine component is represented by an adenocarcinoma/carcinoma which can show different degrees of differentiation, while the neuroendocrine component is represented by a differentiated neuroendocrine tumor which can show grade 1 (NET G1) or grade 2 (NET G2) differentiation, in line with the criteria proposed in the recent 2010 WHO classification of tumors of the gastrointestinal tract [4]. Unlike high grade MANECs, the exocrine component is biologically more aggressive than the neuroendocrine one. Amphicrine carcinoma represents a peculiar tumor in which exocrine and neuroendocrine features are co-expressed by the same neoplastic cells, which show a divergent differentiation demonstrable with immunohistochemical or electron microscopic techniques.

#### 2.2.1. Mixed Adenocarcinoma-Neuroendocrine Tumor (NET)

**Figure 8:**
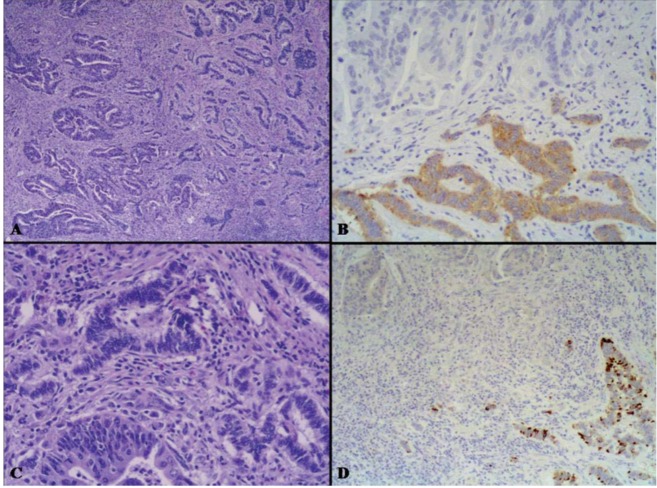
The intermediate grade MANEC category includes mixed adenocarcinomas-NETs which are formed by areas of adenocarcinoma and areas of grade 1 or 2 NET. In (**A**) the tubular adenocarcinoma component is evident on the left, while the NET showing a trabecular pattern of growth is observed on the right. The neuroendocrine component is immunoreactive for synaptophysin (**B**) and is characterized by well differentiated neuroendocrine cells without significant atypia growing in trabecular structures (**C**) negative for Ki67, which is conversely well expressed in the adenocarcinomatous areas (**D**).

**Figure 9:**
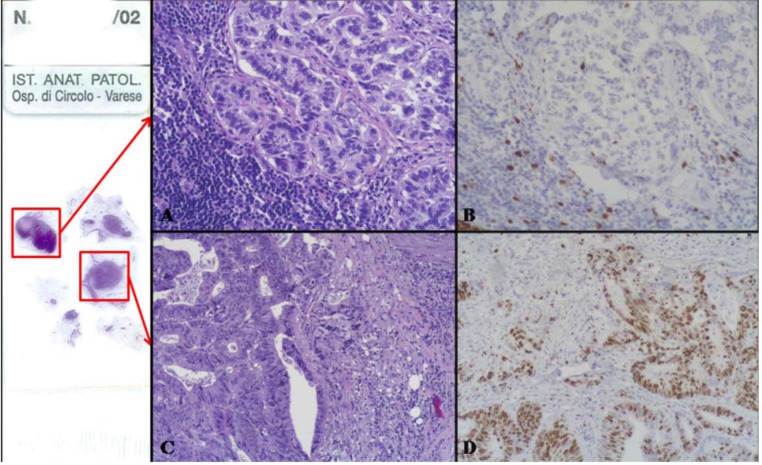
On the left, there is a slide with lymph nodes isolated in the perirectal fat tissues of the tumor shown in Figure 7. In one lymph node there was a metastasis from the NET component (**A**) which shows very low Ki67 index (**B**). In another lymph node the metastasis was from the adenocarcinoma (**C**) which shows a high Ki67 proliferative index (**D**).

Mixed adenocarcinoma-NET is a composite tumor formed by areas of tubular, papillary or mucinous adenocarcinoma and areas of grade 1 or 2 NET in both primary and metastatic sites (Figure 8 and Figure 9). Other names used to diagnose such tumors include mucin-producing carcinoid [47], composite carcinoid-adenocarcinoma [48,49], composite carcinoid tumor [50], mixed adenocarcinoid tumor [51], and composite glandular-neuroendocrine mixed tumor [30]. These neoplasms have been reported in the esophagus [47,52], stomach [18,48,53,54,55], ampulla of Vater [49,56], ileum [51], and large bowel [30,50,57,58,59,60,61]. To date, about 60 cases have been reported in the literature. Tumors occur with a slight prevalence in men at an average age of about 65 years (range 32–87 years). In the stomach, tumors are almost equally distributed in the body and in the antrum, with a prevalence of polypoid lesions measuring in size from 1.5 to 10.5 cm [48,55,62,63,64,65]. All parts of the large bowel from the cecum to the rectum can be involved [50,57,58,60,61,66]. The majority of colorectal neoplasms are large (5 to 7 cm in size) and appear as annular constricting neoplasms. Histologically, most tumors are composed of moderately differentiated tubular, papillary, or mucinous adenocarcinoma and areas consisting of solid nests, sheets or trabeculae of well differentiated neuroendocrine cells, traditionally reported as carcinoid and now classifiable as NET G1 or NET G2 (Figure 8) [4]. Transitional aspects between the two components, although not prominent, are observed in practically all cases. The exocrine differentiation (intestinal or gastric) is defined by mucin production and the presence of exocrine tumor markers such as carcinoembryonic antigen (CEA), epithelial membrane antigen (EMA) and specific mucins [49,50,57]. The neuroendocrine component stains with the usual neuroendocrine immunohistochemical markers [5,48,65,67]. At least five adenocarcinoma-NETs of the stomach were associated with autoimmune chronic atrophic gastritis and with multiple proliferative neuroendocrine lesions such as intramucosal enterochromaffin-like (ECL) cell micro-NETs (microcarcinoids) (Table 2) [48,65,68,69,70]. Some ileal [51] and cecal [60] mixed neoplasms arose in a background of long standing IBD and an esophageal MANEC with a NET component was associated with Barrett esophagus [52]. An unusual variant of MANEC with a NET component is the “composite glandular and endocrine tumor with pancreatic acinar differentiation” [71], which can develop in the stomach [71,72,73,74,75,76] or the ampulla of Vater [77]. The predominant histological pattern of this neoplasm consists of solid nests of polygonal neuroendocrine cells surrounded by numerous vessels. Small acinar laminae punctuate the nests. Another component is represented by well differentiated adenocarcinomas forming glandular or ductal structures, lined by cells resembling gastric foveolar cells and positive for cytokeratin 7, CEA and MUC2. The solid areas are positive for pancreatic acinar markers which overlap with neuroendocrine markers. The main clinico-pathologic features of these gastric neoplasms are reported in Table 3.

Most MANECs with a NET component and especially those located in the large bowel appear at the time of diagnosis as tumors at an advanced stage with deep wall invasion. In addition, lymph node metastases were registered in about half of the cases so far reported, and distant metastases were reported in some cases [48,58,65,78]. In some of the metastatic cases the presence of a neuroendocrine component in lymph node or liver foci was reported [51,65]. Because of the scarcity of follow-up data and the rarity of these tumors, the prognosis of patients with MANECs with a NET component needs to be better defined with further study.

**Figure 10:**
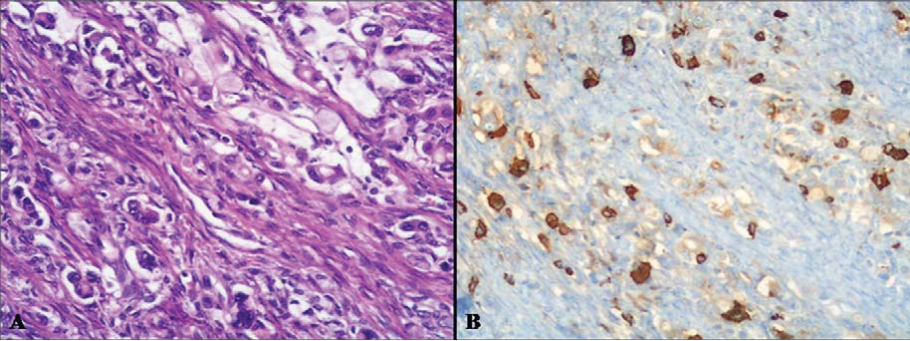
Mixed poorly cohesive carcinoma-NET is a combined neoplasm composed of noncohesive signet ring cells diffusely admixed with neuroendocrine cells (**A**), which are easily detectable using chromogranin A antibody (**B**).

In the group of mixed adenocarcinoma-NET can also be included mixed poorly cohesive carcinoma-NET, which represents a combined neoplasm composed of noncohesive signet ring cells or other cellular variants diffusely admixed with neuroendocrine cells (Figure 10). Other denominations of this tumor include: scirrhous argyrophil cell carcinoma [79], signet ring cell carcinoid [80], and adenocarcinoma ex goblet cell carcinoid [81]. This tumor has been detected in the stomach [70,79], duodenum [82], ampulla of Vater [83], gallbladder [80], appendix [81,84], and colon [85].

**Table 2:** Clinico-pathologic features of adenocarcinoma-neuroendocrine tumor (NET) in autoimmune chronic atrophic gastritis.

Parameter	Caruso *et al*. [48]	Pasquinelli *et al*. [65]	Adhikari *et al*. [68]	Ronellenfitsch *et al*. [69]	Nugent *et al*. [70]
**Age (year)**	53	79	52	53	64
**Sex**	F	F	M	M	F
**Site in the stomach**	Body	Body	Body	Body	Body
**Satellite NE micronodules**	Present	Present	Present	Present	Present
**Carcinoma type**	Intestinal	Signet ring cell	Intestinal	Intestinal	Signet ring cell
**Chronic atrophic gastritis**	Present	Present	Present	Present	Present
**Metastasis**	Absent	Liver, Bone	Liver, LN	Absent	LN
**Prognosis**	Alive	DOD	Alive	Alive	Alive

F: female; M: male; NE: neuroendocrine; LN: lymph node; DOD: died of disease.

**Table 3:** Gastric composite glandular and neuroendocrine tumors with pancreatic acinar differentiation.

	Age (yeras)	Sex	Site	Size (cm)	Associated non acinar component	LN metastasis	Distant metastasis	Follow-up (months)
Fukunaga *et al*. [72]	77	F	Fu	1.2	Solid, glandular	No	No	AFD (7)
Sun and Wasserman [73]	86	F	A	5	Solid, signet ring	No	No	nk
Jain *et al*. [71]	41	F	B	1.5	Solid, glandular	Present	No	AFD (24)
	61	M	Fu	3.3	Solid, glandular	Present	Liver	Lost
	72	M	B	nk	Solid, glandular	No	No	DOC (4)
Ambrosini-Spaltro *et al*. [74]	52	M	A	4	Trabecular	No	No	nk
Kusafuka *et al*. [75]	59	M	B	6	Solid	Present	Liver peritoneum	DOD (3)
Capella *et al*. [76]	67	F	A/B	6	Solid	Present	No	POD
	49	M	B/Fu	15	Diffuse, glandular	Present	Liver	DOD (14)
	55	M	A	8	Diffuse, signet ring	Present	No	DOD (9)
	91	M	B	3,7	Glandular	Present	Liver	DOD (2)
	58	M	A	5,5	Diffuse, signet ring	No	No	NED (9)

F: female; M: male; Fu: fundus; A: antrum; B: body; AFD: alive free of disease; DOC: died of other cause; POD: died post-operatory death; DOD: died of disease; nk: not known.

In the stomach this tumor, according to Tahara *et al*. [79], appears at a mean age of 53 years and in the majority of cases involves the entire stomach with a pattern of linitis plastica. Appendiceal tumors are reported to be more frequent in women than in men [81]. The average age at presentation in one study was 50.5 years (range from 31 to 80 years). In the majority of cases tumors were grossly well evident as diffusely infiltrating indurated masses that invaded the adjacent cecum and peritoneum.

Histologically, gastric neoplasms appear as diffuse type carcinomas with signet ring cells, often lying in an abundant fibrous (desmoplastic) stroma. The exocrine cells are well demonstrated by mucin staining, while the neuroendocrine cells, interspersed among signet ring cells, are well detected with the aid of Grimelius’ silver stain or chromogranin A staining and represent the vast majority of neoplastic cells [79].

Immunohistochemical studies reveal scattered neuroendocrine cells positive for chromogranin A (Figure 10B) and synaptophysin. The goblet and signet ring cells express CEA [81,84]. The majority of patients with mixed diffuse carcinoma-NET of the stomach showed metastases to regional lymph nodes and neuroendocrine cells were also detected within metastatic foci in some cases [79]. Cases with generalized gastric involvement have an ominous prognosis, with all patients dying within 10 months after surgery. Localized forms have a better rate of survival [79]. Twenty-three of 26 cases of adenocarcinomas ex goblet cell carcinoids, signet ring cell type, of the appendix presented T4 disease and the remaining three presented T3 disease at diagnosis. Eleven had nodal metastases and 23 had distant metastases. The 5-year disease-specific survival was 36% [81].

#### 2.2.2. Amphicrine Carcinoma

The first description of amphicrine cells was by Feyrter in his classic description of the “diffuse endocrine epithelial system” [86]. Successively, Ratzenhofer described the presence of cells showing sub-nuclear argentaffin granules and apical mucin vacuoles in rabbit gastric mucosa [87] and suggested the term “amphicrine” to describe such cells. In addition, he also reported the presence of amphicrine cells in tumors of the stomach, appendix and colon [88]. Amphicrine carcinomas are predominantly composed of these characteristic cells which show a bivalent differentiation such as mucus and neuroendocrine granules within the cytoplasm of the same cells. Amphicrine carcinomas are extremely rare. At least 4 cases have been reported in the stomach [54,89,90].

## 3. Mixed Adenoneuroendocrine Tumors (MANETs)

As explained previously, this category includes neoplasms formed by well differentiated neuroendocrine and exocrine cells which behave in an indolent manner.

### 3.1. Adenoma-Neuroendocrine Tumor *(NET)*

**Figure 11:**
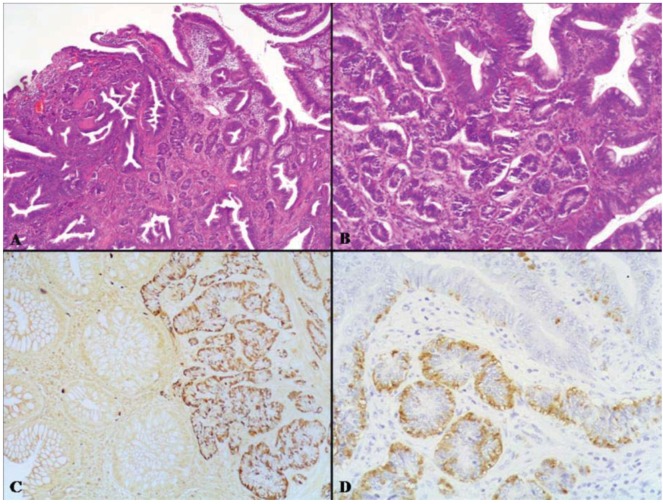
Adenoma-neuroendocrine tumor is a very rare neoplastic lesion containing both an adenomatous (top) and a NET (central portion) component (**A** and **B**, lower and higher magnification, respectively). The NET component is positive for Grimelius’s silver stain (**C**) and Chromogranin A (**D**).

This is a very rare neoplastic lesion containing both an adenomatous and a NET component (Figure 11). This tumor has also been referred to as glandular-carcinoid tumor [9] and has been reported in the colon and rectum and terminal ileum [9,10,91]. Four colorectal cases have been described in two studies [9,10] and we examined such a tumor, located in the rectum of a 45-year-old man. The patients were from 45 to 80 years of age. Macroscopically, neoplasms appear as polyps ranging in size from 1.5 to 3 cm. Histologically, they consist of an adenomatous component either of tubular or villous type, with low- or high-grade dysplasia, and of a NET (carcinoid) component. In some cases the neuroendocrine cells intermingle with the adenomatous glands forming a combined tumor, while in other cases the two components appear to arise as separate lesions juxtaposed to one other (collision tumor). In the case of composite tumors, the polyp had the neuroendocrine component in the center, whereas the adenomatous component occupied most of the periphery of the polyp. Neuroendocrine cells form solid nests and are of small size. They have nuclei with stippled chromatin, lacking significant atypia or mitotic activity. The neuroendocrine component was reported to be argyrophil, chromogranin A and/or synaptophysin positive [9,10]. In some cases positivity for serotonin, somatostatin, and glucagon has been detected [9]. Tumors were limited to the mucosa-submucosa and were removed by endoscopic polypectomy or trans-anal excision. Their prognosis seems excellent (Table 4) because no evidence of tumor recurrence was found in any of the cases so far reported [9,10]. The morphological and prognostic features of these rare neoplasms seem to suggest that they are low grade malignant tumors. They represent an entity not included in the 2010 WHO classification [4]. It is reasonable to propose for this specific category the term “mixed adenoneuroendocrine tumor” (MANET).

**Table 4:** Clinico-pathologic features of adenoma-neuroendocrine tumors.

	Varghese *et al* [91]	Moyana *et al*. [9]		Lyda *et al*. [10]	
		Case 1	Case 2	Case 1	Case 2
**Age (year)**	76	68	75	37	80
**Sex**	M	F	F	M	M
**Site**	Terminal ileum	Cecum	Rectum	Rectum	Ascending colon
**Size (cm)**	4	1.5	2	1	3
**Pathology**	TA-NET	VA-NET	Adenoma-NET	VA-NET	Adenoma-NET
**Follow-up**	Nk	Alive	Alive	Alive	DOC

M: male; F: female; TA: tubular adenoma; VA: villous adenoma; NET: neuroendocrine tumor, Nk: not known; DOC: died of other cause.

## 4. Pathogenesis and Molecular Findings

Few studies in the literature have addressed the histogenetic issue of MANECs and most have reported different findings and controversial data, thus leaving various histogenetic hypotheses still unconfirmed. Mixed neuroendocrine-glandular tumors may result from either the simultaneous proliferation of multiple cell lineages or the proliferation of stem cells capable of differentiating along multiple cell lineages. The presence in MANECs of amphicrine cells containing within their cytoplasm both neuroendocrine secretory granules and mucin droplets supports the hypothesis of a common precursor stem cell capable of divergent differentiation within an individual neoplastic cell [2,92].

In gastrointestinal MANECs there have been genetic studies applying different techniques (loss of heterozygosity, mutational analysis, and others). Data on gastric and colorectal MANECs indicate a relatively higher frequency of chromosomal abnormalities in the NEC than in the adenocarcinoma component. However, shared LOH at chromosomes 5q, 11q, 17p, and 18q suggest a close genetic relationship and a possible multistep progression from a common precursor lesion [16,25,93].

## 5. Management of MANEC

The optimal strategy of management of MANECs and MANETs is largely unknown, due to the rarity of these neoplasms. When considering treatment, the more aggressive component of MANECs should be taken into account. MANECs containing a well differentiated NET component and an adenocarcinoma component should be treated as adenocarcinomas. MANECs containing a poorly differentiated NEC component should be treated as NECs [94].

## 6. Conclusions

Gastrointestinal MANECs are a heterogeneous group of tumors showing different morphological, clinical, and prognostic features. A correct diagnosis allows one to classify them in different prognostic categories that should be taken into account for the choice of the correct treatment.
